# The uptake of metal–organic frameworks: a journey into the cell

**DOI:** 10.1039/d0cs01414a

**Published:** 2022-06-30

**Authors:** Emily Linnane, Salame Haddad, Francesca Melle, Zihan Mei, David Fairen-Jimenez

**Affiliations:** The Adsorption & Advanced Materials Laboratory (A^2^ML), Department of Chemical Engineering and Biotechnology, University of Cambridge, Phillipa Fawcett Drive CB3 0AS UK df334@cam.ac.uk

## Abstract

The application of metal–organic frameworks (MOFs) in drug delivery has advanced rapidly over the past decade, showing huge progress in the development of novel systems. Although a large number of versatile MOFs that can carry and release multiple compounds have been designed and tested, one of the main limitations to their translation to the clinic is the limited biological understanding of their interaction with cells and the way they penetrate them. This is a crucial aspect of drug delivery, as MOFs need to be able not only to enter into cells but also to release their cargo in the correct intracellular location. While small molecules can enter cells by passive diffusion, nanoparticles (NPs) usually require an energy-dependent process known as endocytosis. Importantly, the fate of NPs after being taken up by cells is dependent on the endocytic pathways they enter through. However, no general guidelines for MOF particle internalization have been established due to the inherent complexity of endocytosis as a mechanism, with several factors affecting cellular uptake, namely NP size and surface chemistry. In this review, we cover recent advances regarding the understanding of the mechanisms of uptake of nano-sized MOFs (nanoMOFs)s, their journey inside the cell, and the importance of biological context in their final fate. We examine critically the impact of MOF physicochemical properties on intracellular trafficking and successful cargo delivery. Finally, we highlight key unanswered questions on the topic and discuss the future of the field and the next steps for nanoMOFs as drug delivery systems.

## Abstract 2

This review critically evaluates the recent advancements in the understanding of endocytosis of nano-sized metal–organic frameworks and the importance of biological context in aiding MOF rational design and synthesis for drug delivery applications.

## Graphical abstract

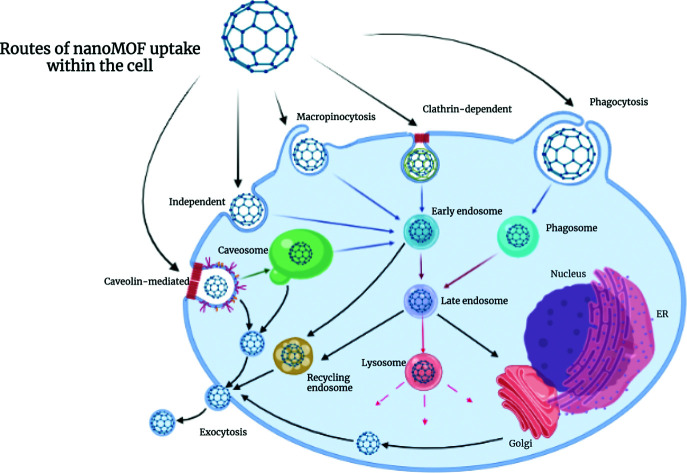

## 1. Metal–organic frameworks and their composites as drug delivery systems

**Fig. 1:**
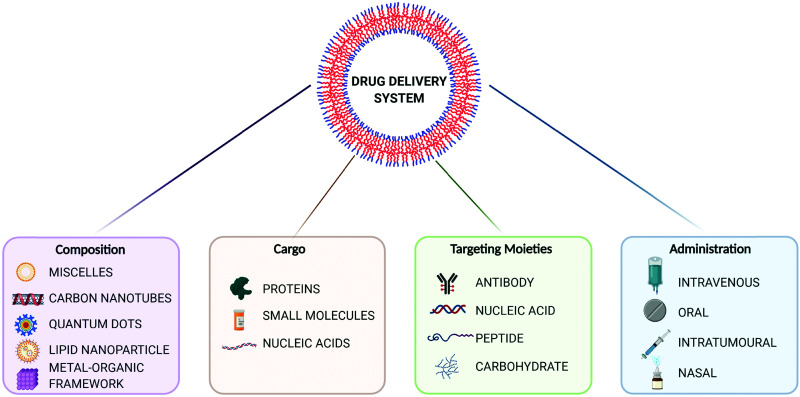
Overview of drug delivery systems (DDS) for precision medicine. Created with BioRender.com.

The evolution of drug delivery systems (DDS) has advanced rapidly since the first sustained release formulation in the 1950s, with novelties bridging the biological, chemical and physical sciences.^1,2^ Current DDS can improve the pharmacological activity and bio-distribution properties of cargo molecules, as well as include cell-specific targeted delivery and stimulus-responsive drug release. Some of the best examples of DDS include organic (*e.g.* liposomes, polymers, micelles and carbon nanotubes) and inorganic (*e.g.* quantum dots) nanoparticles, whereas the recently added metal–organic frameworks (MOFs) can be seen as hybrid materials.^3,4^ The rational selection of an optimal material for particle design and bioengineering is dependent on the type of cargo, the route of administration, and the delivery strategy, which can be carefully designed for a precision medicine approach (Fig. 1). In this scenario, and in particular for the clinical translation of novel DDS, the understanding of the cellular delivery and uptake of nanomaterials remains one of the most pressing issues in the field.

**Fig. 2:**
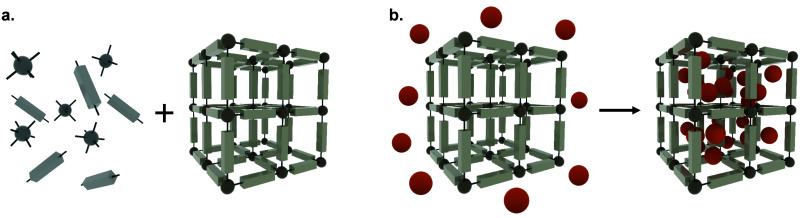
The self-assembly process of metal–organic frameworks (a) The synthesis of metal–organic frameworks composed of metal clusters or ions (dark grey spheres) and organic linkers (light grey units), and (b) the encapsulation of APIs (orange spheres) adsorbed within the porosity.

Metal–organic frameworks (MOFs), also called porous coordination polymers (PCPs), are crystalline, porous materials composed of metal clusters or ions bridged by organic linkers (Fig. 2). They are one of the most exciting developments in recent porous materials science, representing the beauty of coordination polymers. Due to their structural design, MOFs have tunable surface chemistries and pore sizes (from microporous, *i.e.* <2 nm; to mesoporous structures, *i.e.* 2 to 50 nm) and exceptionally high specific surface areas (up to 8000 m^2^ g^−1^).^5–9^ Besides, MOFs offer accessible Lewis acidic metal sites and amphiphilic microenvironments in the porosity that allows for further functionalization, protecting cargos or anchoring groups such as targeting moieties on the external surface of the particles.^6^ Their tunable properties have allowed for a wide range of versatile applications, including gas adsorption and separation, catalysis, and sensing.^10^ Over the past decade, however, MOFs have gained traction as candidates for drug delivery applications,^10–13^ where they have been used to encapsulate different cargos, through biomineralization, physisorption to the internal or external MOF surface, and covalent binding.^8,14^ Indeed, many issues related to the reduced long-term stability of MOFs in energy applications are an advantage for drug delivery, since their degradation *in vivo* after releasing their cargos will avoid their accumulation in the body, therefore minimizing side effects and toxicity.

The first proposed use of MOF nanoparticles (MOF-NP) for drug delivery was laid out by Horcajada and co-workers in 2006 in a proof-of-concept study using two chromium (Cr)-based MOFs: MIL-100 and MIL-101 (MIL = Matériaux Institut Lavoisier). Although MIL-100 and -101 were first studied using toxic Cr as metal, they can also be synthesized using Fe, which is more biocompatible.^15^ These two MOFs demonstrated a high-loading capacity of up to 1.4 g of ibuprofen per gram of MOF and showed controlled drug release over three and six days for MIL-100 and MIL-101, respectively.^15^ Since then, biomedical applications of MOFs, ranging from the delivery of biological gases for wound healing^8,16,17^ to theranostic sensing applications,^18–20^ have gained traction, revealing a wide and diverse scope for these materials. Further developments in synthesis have also led to the possibility of incorporating guest materials, such as inorganic NPs, within MOFs. The resulting MOF-NP composites have enabled the construction of more complex materials with additional functionalities.^12,21–23^ These composites have been investigated in a wide range of applications, from electrochemical sensing, environmental adsorption, and catalysis, to imaging and, again, drug delivery.^12,24–29^ In the latter, MOF-NP composites have presented themselves as promising candidates as both components can be exploited to create a multifunctional material, able to carry different therapeutic modalities or a combination of imaging and therapeutic agents.^12^

### 1.1 Metal–organic frameworks for small molecule delivery

Many of the initial works exploring nanosized MOFs (nanoMOFs) in drug delivery have been done using small molecules, such as proof-of-concept drugs (*e.g.* ibuprofen^30^) and oncological drugs including, doxorubicin, α-cyano-4-hydroxycinnamic acid (α-CHC) and 5-fluorouracil (5-FU).^31–34^ The advancement of synthesis and processing techniques has led to nanoMOFs with higher loading capacities^35^ and prolonged cargo release.^36–40^ For example, although most standard MOFs release drugs in less than 48 h, we have shown how the post-synthetic mechanical or temperature amorphization of MOFs can induce a partial collapse of the pores around the cargo, slowing its release up to several weeks and preventing the ‘burst’ effect – this has been demonstrated in studies with the Zr-based UiO-66 (UiO = Universitetet i Oslo)^36^ and NU-901 (NU = Northwestern University),^38,39^ and the Bi-based CAU-7 (CAU = Christian-Albrechts-University).^38,39^ Over recent years, nanoMOF studies have combined various aspects of treatment, such as photodynamic therapy with controlled small molecule release for a multifaceted approach.^41^ Despite this flexibility and advances, one of the main challenges in the translation of MOFs to clinical use is achieving targeted delivery to enable their uptake into the correct tissue/cells and to avoid off-target or unwanted drug side effects.

### 1.2 Metal–organic frameworks for macromolecule delivery

In the last years, the interest in MOFs as DDS has moved towards their use to carry and deliver macromolecules, including proteins and nucleic acids. Intracellular protein delivery presents a challenge as many proteins are unable to cross the cell membrane due to their size and charge, and are also prone to enzymatic degradation.^42^ Encapsulation of proteins can be a complex endeavour, with MOF stability and protein loading itself being limiting factors. Nevertheless, there has been an increasing number of publications demonstrating protein delivery using nanoMOF formulations. These have included cytochrome *C* delivery for cancer therapy using ZIF-8,^43^ insulin encapsulation for diabetes therapy using the mesoporous, crystalline nanoMOF NU-1000,^44^ and the use of biomimetic ZIF-8 particles for systemic protein delivery using a one-pot synthesis approach.^45^

Looking at other macromolecule-based approaches, nucleic acid-based therapeutics such as small interfering RNA (siRNA), artificial microRNAs (amiRs), messenger RNA (mRNA) and antisense oligonucleotides (ASOs) are versatile modalities. They allow a precision-medicine approach through selective inhibition *via* gene-specific targeting. These molecules act by interfering with cellular processes such as gene translation, mRNA splicing, transcription, and epigenetic regulation.^46^ Probably, the most well-known and timely nucleic acid-based therapeutics – not associated with MOFs – are the vaccines recently presented by Pfizer/BioNTech and Moderna for COVID-19.^47^ Looking forward, despite the promise of RNA-based therapies, several barriers are preventing their broader clinical translation.^48^ Effective internalization of RNA-based molecules is hindered by *in vivo* factors including RNA degrading enzymes, innate immune pattern recognition toll-like receptors (TLRs),^49^ and rapid clearance from the kidney and liver.^50,51^ Perhaps the biggest challenge in the field of nucleic acid therapeutics is intracellular uptake, as the lipid bilayer forming cell membranes prevents large, charged, RNA-like molecules from entering the cell.^52^ The use of nanoparticles for the delivery of macromolecules across cell membranes is one of the most promising approaches to overcoming these limitations. Different strategies have been investigated in the past, including the use of viral materials^53^ and nano-vehicles such as liposomes, polymeric formulations, inorganic nanoparticles and, more recently, nanoMOFs.^54,55^

Of these therapeutics, siRNA, in particular, has shown promise in treating various genetic diseases as well as many cancers.^56^ They are short (21–23 nucleotide), non-coding, double-stranded RNAs with an antisense active strand that is complementary to a sequence in the target mRNA.^57^ To date, several studies have explored the use of nanoMOFs to deliver siRNA into the cell.^55,58,59^ Recently, we used the mesoporous NU-1000 – a MOF identified for this application using molecular simulations – to deliver an mCherry-targeting siRNA, demonstrating enzymatic protection and intracellular delivery in HEK cells.^55^ In addition, Pan *et al.* demonstrated the ability of ZIF-8 (ZIF = zeolitic imidazolate framework) to protect BCL-2-targeting siRNA from nuclease degradation in MCF-7 and SKOV-3 cells.^59^

MicroRNA (miRNA) is another important RNA-based platform for protein knockdown. Endogenous microRNAs are non-coding RNAs that act as key regulators for a variety of cellular pathways and are often downregulated in diseases.^60^ Lin and co-workers demonstrated the use of miRNA-responsive Zr UiO-68 nanoMOF for the selective delivery of the anticancer drug doxorubicin into two types of cancer cells.^61^ However, the size of the macromolecules employed is larger than that of the windows or cavities of UiO-68, which could limit the long-term protection of the cargo. On a similar note, Hidalgo *et al.* developed a system to transport miRNA-145, an onco-suppressor typically downregulated in cancer, using the biocompatible iron(iii) carboxylate nanoMOFs MIL-100 and -101.^62^ Although the window size of the material is again too narrow for the macromolecule, they demonstrated protection from enzymatic degradation. Further tuning of nanoMOF chemistry and advancement of post-synthetic modification techniques will allow for nucleic acid delivery to be exploited further with this technology.

### 1.3 The intracellular delivery enigma

Despite the great advances of MOFs for drug delivery, one of the main challenges for small molecules, proteins, and nucleic acid delivery using nanoMOFs is their trafficking to the correct cellular compartment, avoiding lysosomal degradation and/or exocytosis. This understanding is critical because these cellular events will ultimately determine the final fate of the MOF and its’ contents. As such, and often ignored, the questions on understanding the pathways of nanoparticle internalization and the endosomal escape mechanisms are key for successful cargo delivery and their clinical translation. Over recent years, the evaluation of nanoMOFs and their composites for drug delivery applications has advanced towards their *in vitro* pharmacology and toxicology, especially in relation to oncological malignancies.^4,8,12^ Although there are some excellent reviews for MOFs as DDS,^63–65^ to date, there has been no systematic review in the field of nanoMOF cellular uptake. Herein, this review covers the recent advancements in understanding the MOF journey inside the cell, mechanisms of uptake, and the importance of the biological context in the final fate of a nanoMOF. We critically examine the impact of material composition and external surface chemistry on intracellular trafficking and successful cargo delivery. Finally, we highlight key unanswered questions and discuss the future of the field and the next steps that are required in the proposition and translation of nanoMOFs as drug delivery systems.

## 2. Pathways of intracellular uptake and trafficking of MOFs

**Fig. 3:**
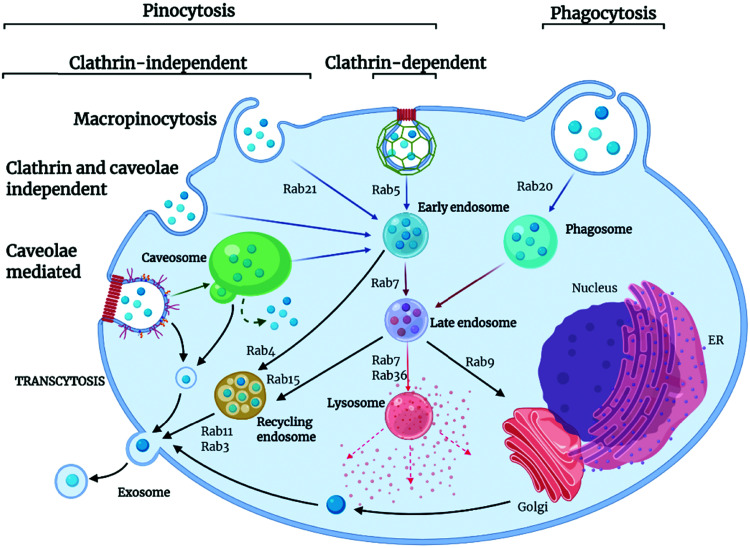
Pathways and routes of endocytosis reported for nanoMOFs. There are several key routes which nanoMOFs may utilise to enter cells; clathrin- and caveolae-mediated endocytosis, macropinocytosis, clathrin and caveolin independent pathways and phagocytosis. These are mediated by the Rab protein family, small guanine triphosphatases (GTPases) that act to regulate pathways of endocytic trafficking. The final fate of the MOF is dependent on many factors including mechanism of uptake, cell type, MOF composition and API. The MOF may travel to the cytoplasm, undergo degradation in the lysosome, be trafficked out of the cell *via* the recycling pathway in exosomes or traffic to the cell nucleus. Created with BioRender.com.

Extracellular ligands, membrane proteins and lipids are transported into the cell through a highly conserved and tightly regulated energy-dependent process, termed endocytosis. Endocytosis is crucial for cell functions such as cell signalling, motility, and uptake of nutrients. In simple terms, endocytosis generates small membrane-bound vesicles (around 50 to 150 nm in size) that can transport cargoes within the cell to different destinations. Of course, this is particularly relevant for nanoMOF internalization and drug delivery applications. The uptake occurs through various mechanisms dependent on cell type, biological context, and cargo being transported, with some cargo being recycled and others marked for degradation within the cell (Fig. 3).^66^ The movement of these vesicular structures is mediated by proteins from the Rab GTPase family, which, when active, regulate the formation, movement, and membrane fusion processes for successful membrane trafficking.^67,68^ Typically, vesicle trafficking involves various organelles within the cell, including the early, late and recycling endosomes, autophagosomes, lysosomes, and the Golgi apparatus. Endocytosis pathways themselves are highly regulated and complex, involving spatiotemporal coordination and cellular signaling pathways. Importantly, these endocytic pathways are well established as the main mechanisms for nanomaterials uptake into cells.^69,70^ Whilst many different endocytic pathways have been reported in eukaryotic cells, the most well-documented pathways of endocytosis include clathrin and caveolae-mediated, clathrin and caveolin independent pathways, macropinocytosis and phagocytosis (Fig. 3).

Clathrin-mediated endocytosis is named after the protein clathrin, a tri-skeleton protein structure formed of heavy and light protein chains, although, in addition to clathrin, there are over fifty other proteins involved in this pathway of trafficking.^71^ Clathrin-mediated endocytosis is utilized for the transport of molecules, such as iron-bound transferrin, within the cell. This pathway begins when cytoplasmic proteins assemble at the cell membrane and recruit other protein components to form clathrin-coated pits, which are composed of adaptor proteins such as AP2 complex. Once assembled, the actin module forms, composed of regulatory components and actin filaments. A scission process then takes place, which constricts the membrane, producing the vesicle. The uncoating process then releases the vesicle, allowing further trafficking to the endosome.^72^ Caveolin-mediated transport is a cholesterol-dependent pathway occurring in the lipid raft membrane domain of the plasma membrane, responsible for the uptake of albumin as well as viruses and toxins. Caveolin proteins are able to undergo oligomerization on the cell membrane, forming a membrane invagination called caveolae. These caveolae can bud off from the membrane and traffic to endosomes. However, other pathways have also been reported whereby caveolae traffic to caveosomes or caveolin-1 positive endosomes, thus avoiding the endolysosomal pathway.^66^

There are also several additional endocytic pathways identified termed clathrin- and caveolin-independent pathways. Such pathways include glycosylphosphatidylinositol-anchored proteins (GPI-AP)-enriched early endosomal compartments (GEECs). GEECs are proposed to result from the fusion of uncoated tubulovesicular clathrin-independent carriers (CLICs). Cell-surface proteins such as MHC I and Tac, the interleukin-2 (IL-2) receptor α-subunit, are internalized in a dynamin- (and clathrin)-independent manner into an ARF6-positive tubular endosomal system, which is distinct from the clathrin-dependent cargo-containing endosomes. Flotillin proteins also are shown to have a function in an independent process. They are localized to small puncta in distinct membrane domains and are required for the induction of membrane invaginations.^73^

Other well-documented routes of endocytosis include macropinocytosis, a non-receptor mediated process used by the cell for internalization of soluble materials and phagocytosis, receptor-mediated engulfment of large particles such as cell debris and bacteria. Macropinocytosis, also known as “cell drinking”, refers to the internalization of extracellular materials such as fluids, solutes, and smaller particles through a fluid phase uptake. It is initiated through signalling molecules such as growth factors, kinases, and integrins, which can trigger changes in the membrane, causing the formation of actin ruffles, which then form protrusions to engulf the extracellular materials.^74^ Internalized material is then incorporated into vesicles termed macropinosomes, which are usually large (around 200 nm to 5 μm) and can traffic into the cytosol. Phagocytosis is the engulfment of large materials for their degradation within the cell, commonly described as “cell eating.” It is the most commonly used pathway in immune cells to destroy unwanted pathogens and cell debris.^66^ Phagocytosis is distinct from other pathways of endocytosis. Here, membrane protrusions form around the materials to be engulfed, and the internalized materials are transported for degradation into the phagosome. Phagocytosis is associated with opsonization, a mechanism by which serum proteins mark a substance to be cleared by immune cells. Opsonization is responsible for the internalization and degradation of nanomaterials *via* this pathway, which will be discussed in more detail later in this review.^75^

## 3. Impact of nanoMOF physicochemical properties on cell uptake

The cellular uptake of nanoMOFs can be evaluated in relation to their physicochemical properties, including particle size, shape, and surface chemistry. Despite the high number of publications involving MOFs, a very small fraction is devoted to their shaping,^76^ and even less to their biologically-adapted formulation. Studying the interaction between morphology and the *in vivo* environment is indeed complex. Firstly, for intravenous injection of the material, it is important to take into consideration the size of the small capillaries in the bloodstream, which require a nanoparticle MOF diameter of less than 200 nm for free movement within the vascular system.^8^ Also, size plays an important role in splenic and renal clearance: particles larger than 200 nm in diameter are cleared by the reticuloendothelial system, whereas those smaller than 10 nm are cleared by renal filtration.^77^ Moreover, particles smaller than 250 nm are reportedly more likely to extravasate through leaky endothelium *via* the enhanced permeability and retention (EPR) effect, a feature that is often described as a principle for nanomedicine tumour targeting including in nanoMOF papers.^78^ However, EPR principle has been recently challenged in the field and remains controversial, and nanoMOF studies would benefit from further investigation of this phenomenon.^79^ Both particle size and shape determine the interaction between the material and the *in vivo* environment, starting from (i) the biodistribution, (ii) the ability to interface with specific cell membranes, (iii) and the cellular uptake.^80^ At the same time, the external surface chemistry of MOF particles impacts the interaction at the particle-cell interface through electrostatic and steric interactions.^81^ The surface charge of a particle – related to the external surface chemistry – is associated with its hydrophilicity, *z*-potential and colloidal stability, all of which are critical in maintaining successful particle delivery.^82^ Besides, the particle–cell interaction will also depend on the cell line under study. To illustrate the complexities of these properties on nanoMOF uptake, here, we first review papers published on NP-MOFs and their composites in the context of endocytosis routes and in relation to their particle size and shape, and surface chemistry and charge.

**Fig. 4:**
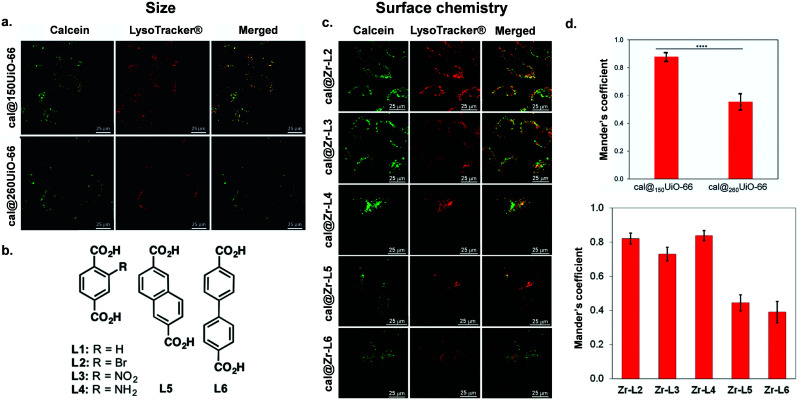
Impact of size and surface chemistry on MOFs internalisation. (a) Confocal microscopy images of HeLa cells incubated with cal@_150_UiO-66 or cal@_260_UiO-66 (green fluorescence, calcein; red fluorescence, LysoTracker-Deep red) for 2 h, reproduced from Orellana-Tavra *et al.*^83^ with permission from Advanced Healthcare Materials, copyright 2016. (b) Organic linkers used to synthesize Zr-based MOFs. (c) Confocal microscopy images of HeLa cells incubated with Zr-based MOFs loaded with calcein (green fluorescence, calcein; red fluorescence, LysoTracker-Deep red) for 2 h.^37^ (d) Both figures show Manders’ overlapping coefficient for all the MOF samples and the lysosome marker (a and b). Image reproduced and adapted from Orellana-Tavra *et al.*^37^ with permission from American Chemical Society, copyright 2017.

Table 1 summarizes the literature on how the particle size, shape and external surface charge and surface chemistry all may impact the cellular uptake route. The first study regarding the endocytosis mechanisms of MOFs was reported by Orellana-Tavra *et al.*, who examined the impact of UiO-66 nanoMOF size on cellular uptake pathways using various pharmacological inhibitors of endocytosis.^83^ They reported trafficking of UiO-66, 150 nm in size, using HeLa (human cervical cancer) cells, mostly through the clathrin-mediated pathway, whilst 260 nm UiO-66 nanoparticles used both the clathrin- and caveolin-mediated pathways (Fig. 4). Additionally, they noted that 150 nm sized particles had a much greater localization with lysosomes compared with the larger particles, indicating that smaller particle-sized UiO-66 are not the most efficient carriers for drug delivery when using HeLa cells.^83^

**Table 1:** Summary of different nanoMOFs, size, shape, charge and modifications on cellular uptake routes

Ref.	Material	Size (nm)	Shape	Surface charge (mV)	Modifications	Cellular uptake route	Cell line
83	UiO-66	150	Spherical	—	—	Clathrin	HeLa
		260	Spherical	—	—	Clathrin- and caveolae	HeLa
37	UiO-66	50 (SEM)	Polyhedron	0.5	—	Clathrin- and caveolae	HeLa
		75 (SEM)	Polyhedron	12.3	—	Clathrin, some caveolae and macropinocytosis	HeLa
		92 (SEM)	Polyhedron	14.2	—	Clathrin	HeLa
		260 (SEM)	Polyhedron	18.9	—	Clathrin	HeLa
		211 (SEM)	Polyhedron	36.5	-Br	Clathrin	HeLa
		129 (SEM)	Polyhedron	42.7	-NH_2_	Clathrin	HeLa
		78 (SEM)	Polyhedron	8.0	Naphthalene-2,6-dicarboxylic acid	Caveolae	HeLa
		115 (SEM)	Polyhedron	−5.8	4,4′-Biphenyldicarboxylic acid	Caveolae	HeLa
84	CpG-UiO-66-NH_2_	105 (TEM)	Spherical	−16	Cytosine–phosphate–guanosine oligonucleotides; calcium phosphate shell	Macropinocytosis	HeLa
85	UiO-66-PEG	160.2 (SEM)	Spherical	—	PEG550	Clathrin	HeLa
		172.9 (SEM)	Spherical	—	PEG2000	Caveolae-mediated	HeLa
39	NU-901	200 (SEM)	Elongated	—	—	Caveolae plus others	HeLa
	NU-1000	150 (SEM)	Elongated	—	—	Caveolae plus others	HeLa
86	cal@(DCA5-UiO-66)	115 ± 48 (SEM)	Spherical	35.3	—	Macropinocytosis	MCF-7
	cal-TPP@ (DCA5-UiO-66)	115 ± 48 (SEM)	Spherical	12.9	Triphenylphos-phonium (targeting mitochondria)	Clathrin	MCF-7
87	MIL88B-NH_2_	937 ± 325 (SEM)	Elongated	11.5	—	Phagocytosis	KUP5
38	CAU-7	195 – 477	—		—	Caveolae-mediated	HeLa
88	PCN-224	30–190	Elongated	—	Folic Acid	Non-specific endocytosis	HeLa
89	Zr-fum H_6_-GFP	182 (DLS) (84 ± 7) SEM	—	—	His-tag peptides	Macropinocytosis	HeLa
90	MIL-100	250 nm (DLS)	—	—	DOPC coating	Clathrin-mediated	HeLa, A431 A549, MCF7, MCF10A THP-1

Some studies have shown that surface modifications (an aspect that will be discussed in more detail in Section 4) affect particle shape, charge and size, which in turn can also influence the route of uptake. For example, Abanades Lazaro *et al.* reported that, through the addition of different lengths of PEG on the external surface of UiO-66 particles, they were more rounded in shape with less-defined edges.^85^ They found that, by coating UiO-66 with PEG2000, they increased its uptake through the caveolin pathway, whereas PEG550 did not show uptake through this route. This difference may be due to the effect of the PEG modification, the impact on the particle shape or a combination of both. Other modifications that impact the size and shape of nanoMOF particles have also been explored. Wang *et al.* synthesized UiO-66 and coordinated cytosine–phosphate–guanosine (CpG) oligonucleotides onto the MOF surface. They showed that the CpG–MOF was selectively inhibited by the macropinocytosis inhibitor EIPA, suggesting that CpG–MOF was internalized mainly *via* macropinocytosis.^84^ Haddad *et al.* reported that, when using triphenylphosphonium (TPP) conjugated in UiO-66 nanoMOFs, they were internalized by clathrin-mediated endocytosis. In contrast, the naked UiO-66 nanoMOF was internalized mostly by clathrin- and caveolae- independent endocytosis this was possibly provoked by the positive charge of the TPP molecule.^86^ Whilst many studies examining MOF uptake have been carried out in cancer cell lines, Durymanavo *et al.* examined the uptake of iron nanoMOF MIL-88B-NH_2_ using the Kupffer liver cell line (KUP5). They showed that the nanoMOFs were internalized *via* phagocytosis.^87^

Taking into account different metals, the Bi-based CAU-7 was reported to enter cells through both clathrin- and caveolin-mediated endocytosis, with a preference for caveolin (58% reduction of internalization upon caveolin inhibition).^38^ However, in this case, CAU-7 nanoparticles were heterogeneous in size, with sizes ranging from 195 ± 12 nm to 477 ± 40 nm, limiting the conclusions of this study as differences in particle size could account for the different routes of uptake. A similar pattern was observed for Zr-based MOFs NU-901 and NU-1000, with *in vitro* studies suggesting that caveolin was the main route of cellular entry.^39^ A noteworthy caveat for any *in vitro* study is that MOFs with unprotected external surfaces can potentially aggregate, which can impact their hydrodynamic size and therefore their routes of internalization and *in vivo* behaviour.^91^ Indeed, this is a fundamental consideration when evaluating nanoMOF data, as particles taken up by clathrin and caveolin mediated endocytic pathways are limited to a maximum size of around 300 nm whereas larger particles (500 nm plus) are mostly internalized *via* macropinocytosis or phagocytosis mechanisms.^92^ Park *et al.* studied the size-dependent cellular response of five samples of Zr-based PCN-224 (a MOF containing a photosensitizer linker, tetrakis (4-carboxyphenyl) porphyrin (H_2_TCPP)), with particle size ranging from 30 to 190 nm in HeLa cells.^88^ Quantitative uptake analysis using inductively coupled plasma mass spectrometry (ICP-MS) with a dose range of PCN-224 (0.5 to 40 μM) showed that different quantities of Zr entered into the cells, suggesting the different sizes of particles indeed resulted in different cellular responses, with 90 nm-PCN-224 showing the highest amount of Zr in cells. They also studied time-dependent cellular uptake of PCN-224 NPs in order to elucidate the kinetics of cellular uptake, showing a plateau around 12 h for each condition. However, despite reporting that uptake of PCN-224 into the cell was thorough endocytosis, no biological study to elucidate uptake pathway was carried out as part of this study. This work also included the synthesis of a series of folic acid (FA)-modified, 90 nm, PCN-224 NPs with stoichiometries of 1/8, 1/4, and 1/2 equivalents of FA to the available Zr binding sites. They tested the targeted photodynamic therapy (PDT) efficacy, with 1/4-FA-PCN-224 showing the highest efficacy. Although all FA modifications provided an enhancement in efficacy compared to the control, the lower potency of 1/8-FA-PCN-224 may be explained by a lower FA density compared to 1/4-FA-PCN-224. On the other hand, the lower enhancement in efficacy of ½-FA-PCN-224 is probably explained by the size expansion of the MOF. Although we will discuss folic acid modifications in more detail later in this review, these results show that, even in the presence of a modification that is able to enhance the cellular uptake of the nanoparticles, it is important to find a balance between external surface functionalization and particle size. Another study by Roder *et al.*, synthesised MIL-88A, HKUST-1 and Zr-fum MOFs functionalised with various oligohistidine-tags (His-Tags) based on a self-assembly process and studied their intracellular uptake in HeLa cells. Whilst MIL-88A and HKUST-1 MOF NPs had very low levels of detectable cell uptake (which the authors speculate was due to larger size particles aggregating) they reported that Zr-fum H_6_-GFP particles were trafficked into the cell *via* macropinocytosis.^89^ A study by Ploetz *et al.* with lipid-coated iron nanoMOFs (MIL-101) showed delivery of iron ions into cancer and immune cell lines *via* clathrin-mediated endocytosis and that acidification of the extracellular pH leads to degradation of MOF nanoparticles inside the cell resulting in cell death by pyroptosis.^90^ Further functional studies exploring the impact of nanoMOF shape, size and surface chemistry and charge on a range of different cell lines will provide a better understanding of the factors affecting cellular uptake mechanisms.

## 4. External surface functionalization

**Fig. 5:**
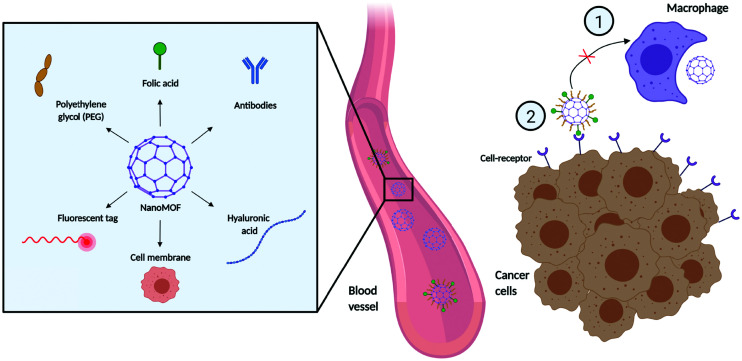
Examples of external surface functionalization techniques in nanoMOF delivery and distribution. Different types of surface modifications can (i) prevent the macrophagocytic clearance: and (ii) improve cell selectivity and targeting. Created with BioRender.com.

Despite recent advances in MOFs for drug delivery, there are still important challenges regarding MOF stability, aggregation in solution, biodistribution, and specific tissue targeting.^93^ Some studies have addressed the modification of the external surface of the MOF to improve their colloidal stability and dispersibility.^94,95^ In terms of targeting, distinguishing between cancer and normal cells presents challenges in cancer treatment, as unwanted off-target effects can lead to unpleasant side effects and damage to healthy cells. This has raised the attention towards new targeting strategies that capitalize on differences in cellular processes and surface markers between cancer and healthy cells. The grafting of different molecules on the MOF external surface can affect the pathways and routes of cellular internalization, endow stealth capabilities, improve the pharmacokinetic and pharmacodynamic properties of the NPs, and provide cell-specific targeting (Fig. 5). This can be challenging in and of itself, as the external surface modification agent and process need to ensure that they, (i) do not interfere with the MOF structure, (ii) improve the stability of the material in biological environments, (iii) and do not interfere with the entrapped molecule.^96^ Compared to other classes of nanoparticles, MOFs offer a distinct advantage regarding external surface functionalisation strategies. The presence of unsaturated metal sites and linker sites (*e.g.* carboxylic acid) ensures that MOFs can be coordinated or covalently bound by various molecules, which can improve their cellular uptake and behaviour in biological systems.^97^ The functionalization of the external surface of a MOF can be done either by post-synthetic modification *via* coordination of molecules to free metal sites or *e.g.* click chemistry on the organic ligands, or through the incorporation of molecules during the synthesis process using modulators as capping agents.^85,98,99^ The possibilities for functionalization are extremely diverse, with agents ranging from surfactant coatings, PEG, and other materials that aid uptake (*e.g.* hyaluronic acid) to antibodies for targeting.

### 4.1 PEGylation

One of the most prevalent external surface modifications in the field of nanomedicine is the addition of polyethylene glycol (PEG) to the surface of NPs. PEG is a hydrophilic coating that can modify the material performance by increasing steric hindrance, which prevents particles from aggregating and reduces serum protein binding to the particle surface.^100^ This decreased protein binding, in turn, reduces the macrophage uptake, offering the NPs stealth-like properties and, ultimately, increasing their stability and improving their pharmacokinetic/pharmacodynamic properties.^101,102^

One of the first studies using PEGylation in MOFs described the grafting of different PEG chain lengths to the external surface of UiO-66 through click-chemistry.^85^ As PEG chain length increased, the MOF particle edges became less defined and their shape became more rounded. Besides, the MOF size increased from 146.6 ± 29.3 nm for non-PEGylated UiO-66 to 160.2 ± 26.9 nm and 172.9 ± 36.8 nm for UiO-66-PEG550 and UiO-66-PEG2000, respectively, where 550 and 2000 represent the molecular weights of the PEG chains. As both the particle size and external surface chemistry can impact interactions with the cell membrane, this has implications for cellular uptake. During the *in vitro* studies with calcein-loaded particles, the non-PEGylated UiO-66 and UiO-66-PEG550 particles were trafficked mainly through the clathrin pathway, with a minor contribution for macropinocytosis. In contrast, the particles with the largest chain, UiO-66-PEG2000, trafficked through clathrin, caveolin and macropinocytosis routes. When examining the intracellular distribution using confocal fluorescence microscopy, pristine and PEGylated UiO-66 were found in the lysosomes after 2 hours of incubation. This agrees with their observed entry through clathrin-mediated pathways. However, in the case of UiO-66-PEG2000, the NPs were also found outside the lysosomes, confirming the alternative and more interesting uptake mechanisms. Another study functionalized the surface of Fe-based MIL-101 nanoMOFs through grafting PEG chains and alendronate onto a dextran backbone (DEX-ALN-PEG).^30^ This is particularly important because MIL-101(Fe) is not stable in water and the PEGylation can enhance stability. Besides, the experiments with human serum albumin proved that the DEX-ALN-PEG coating reduced the adsorption of proteins to the external surface compared to the uncoated MIL-101(Fe). Using ICP-MS and cell staining to evaluate the cell internalization kinetics, they showed that the uptake of the coated particles using the macrophage cell line J774A.1 was significantly reduced, as expected. In addition, they obtained a direct relationship between the length of the PEG coating and the level of uptake, with the larger PEG molecules being associated with lower macrophage uptake.

An important issue of MOFs when exposed to phosphate-based and other buffers is that they can often become unstable. Specifically, the phosphate ions in buffers such as PBS – ubiquitous in pharmaceutical applications – coordinate with the metal clusters, breaking down the MOF structures. Chen *et al.* developed MOF coatings for a series of Zr-based MOFs (PCN-128, 222, NU-901, MOF-808, UiO-66) to protect MOFs from the phosphate-ion degradation.^103^ In this case, the coordination to the metal cluster was done through a phosphate head connected to a PEG chain (mPEG-PO_3_). The phosphate head provided stronger binding than previous designs based on *e.g.* click-chemistry, resulting in MOFs with excellent colloidal and hydrochemical stability in different solvents and buffers. Further studies of different nanoMOFs with the mPEG-PO_3_ coating in other physiological buffers would provide additional insight into MOF stability *in vitro* and *in vivo* experiments. In terms of efficacy, Chen *et al.* also carried out *in vitro* studies that showed that the presence of mPEG-PO_3_ reduced the cytotoxicities of the studied nanoMOFs at high concentrations, avoiding the burst release of the preloaded cancer drug doxorubicin. Moreover, they studied the possibility of long-term MOF storage – by freeze-drying the PEGylated MOF solutions, demonstrating they could redisperse them without producing any aggregation. This is particularly important to increase the MOFs shelf-life and their translation to the clinic.

### 4.2 Folic acid

The folate receptor is a glycosylphosphatidylinositol-anchored cell surface receptor that is overexpressed on the vast majority of cancer tissues, including epithelial, ovarian, cervical, breast, lung, kidney, colorectal, and brain tumours, while its expression is negligible in healthy tissues and organs.^104,105^ As such, many efforts have focused on modifying the surface of MOFs and other NPs with folic acid. Wang and co-workers used folate and chains of PEG to functionalize the external surface of a newly synthesized bioMOF (CaZol nanoMOF) prepared from calcium and the therapeutic agent zoledronate (Zol), a widely used anti-resorptive agent for the treatment of cancer bone metastasis.^106^ The folate-targeted lipid coating facilitated the endocytosis of the CaZol nMOFs in FR-overexpressed cancer cells, allowing the nanoMOFs to control the release of encapsulated Zol in mildly acidic endosomes. Due to the increased cellular uptake and prolonged drug release kinetics, the Fol-targeted CaZol nanoMOFs showed higher efficiency than small-molecule Zol at inhibiting cell proliferation and inducing apoptosis in FR-overexpressing H460 and PC3 cancer cells *in vitro*. Moreover, quantitative *in vivo* antitumor activity studies in H460 and PC3 xenograft tumour-bearing mice showed that Fol-targeted CaZol nanoMOF improved the direct antitumor efficiencies of Zol by approximately 80%, something that did not work when using the non-targeted CaZol.^106^ Another study incorporated PEG and folate to functionalize the ZIF-8 in order to co-deliver *p*-glycoprotein inhibitor verapamil (VER) and the antitumoral doxorubicin hydrochloride (DOX).^107^ The methoxy poly (ethylene glycol)-folate (PEG-FA) used to stabilize the (DOX + VER)@ZIF-8 system allowed prolonged circulation and active targeted delivery.

Building on this, Abanades Lázaro *et al.* assessed different protocols for external surface functionalization of Zr-MOF UiO-66 nanoparticles by (i) direct attachment of biomolecules (such as folic acid (FA) and biotin (Biot)) introduced as synthesis modulators, (ii) “click-modulation”, covalently attaching polymers (poly(ethylene glycol) (PEG), poly-l-lactide, and poly-*N*-isopropylacrylamide), and (iii) external surface ligand exchange to post synthetically coordinate FA, Biot, and heparin to UiO-66.^108^ In their work, they used fluorescence-activated cell sorting (FACS) to study the endocytosis efficiencies and pathways of the calcein-loaded (cal), surface-modified NMOFs when incubated with HeLa cervical cancer cells. First, they showed that cal@UiO-66 uptake efficiency was higher than that of free calcein, proving the validity of NMOFs as carriers to internalize cargoes that are not able to cross the cell membrane by themselves. Thanks to the overexpression of the folate receptor on HeLa cell membrane, cal@UiO-66-FA showed the highest internalization, followed by cal@UiO-66-Hep, cal@UiO-66-L2, and cal@UiO-66-L1-PolyLact. cal@UiO-66-Biot was instead poorly internalized by HeLa cells, showing that biotin coating might not be desirable to enhance NMOF cell internalization. Studies across additional cancer cell panels would provide more robust insights into if this phenomenon is cell line specific to HeLa cells or a general result of biotin coating impacting nanoMOF uptake within the cell. The use of inhibitors for the different endocytosis routes showed how cal@UiO-66-FA was mainly internalized through caveolae-mediated endocytosis. As described above, this mechanism is desirable for efficient treatment, as the nanoMOF can potentially escape the early endosome, avoiding lysosome degradation and facilitating faster drug release in other cellular locations such as the cytosol. These results suggest that the drug-loaded UiO-66-FA samples have the potential to be efficient therapeutic DDSs. Subsequent studies of anticancer drug dichloroacetate (DCA)-loaded materials showed that FA-coated MOFs exhibit selective cytotoxicity towards immortalized cell lines HeLa (cervical) and MCF-7 (breast cancer) cells, without adversely affecting the proliferation of HEK293 (immortalized cell line derived from human embryonic kidney cells, macrophages (J774)), and peripheral blood lymphocytes (PBLs). This is possibly due to the overexpression of the FR on the surface of cancer cells and a preference for desirable caveolae-mediated endocytosis, although, without controlling particle size, it is difficult to fully account for differences in uptake patterns that could be size-dependent. As described in the previous section, we have seen how particle size is important for cell uptake. Nevertheless, this work highlights the significance of MOF chemistry, surface functionalization and the importance of cell internalization pathways in the application of MOFs for drug delivery.

Some complex design combinations have also been reported. Chowdhuri *et al.* developed a magnetic nanoscale MOF by incorporation of Fe_3_O_4_ nanoparticles into the porous isoreticular MOF IRMOF-3.^34^ On the external surface of the system, they conjugated two molecules, folic acid and the fluorescent molecule rhodamine B isothiocyanate (RITC) for imaging by activation of FA followed by addition of Fe3O4@IRMOF-3 using NHS/EDC chemistry. They then loaded the hydrophobic anticancer drug paclitaxel in the Fe_3_O_4_@IRMOF-3/FA complex, showing its high efficiency by targeting and killing HeLa cells. Another study anchored functional folic acid (FA) molecules onto the Zr_6_ clusters of two zirconium-based MOFs, MOF-808 and NH_2_-UiO-66 through the terminal carboxylate of FA molecules, substituting the original formate or terminal OH ligands,^109^ loaded the anticancer drug 5-fluorouracil (5-FU) and studied the different loading and release behaviours at different pH; the higher release took place at pH 5.5. Using confocal laser scanning microscopy and calcein/propidium iodide test, they confirmed that HeLa cells incubated with 5-FU@FA-NH_2_-UiO-66 and 5-FU@FA-MOF-808 were effectively killed compared to the non-folate functionalized MOFs, confirming that the changes in the surface chemistry were responsible for the intracellular delivery. Importantly, they suggested a combination of phagocytosis and receptor-mediated endocytosis as responsible for the uptake. Here, further *in vitro* studies including receptor-MOF interactions would be interesting to understand the mechanism of on-target internalization and the consequent downstream effect. Overall, these preliminary studies show the potential of FA coatings for nanoMOF delivery. However, full characterization of expression of FR across cell models through techniques such as immunofluorescence and flow cytometry would help to correlate uptake with FR expression levels.

### 4.3 Hyaluronic acid

Hyaluronic acid is a linear mucopolysaccharide that makes up part of the extracellular matrix in the body and, due to its reported biocompatibility and stealth capabilities, it has been widely studied for cancer-related nanomedicines.^110–112^ Besides, hyaluronic acid shows high-affinity binding to certain cell surface receptors, including CD44^113^ – an 85 kDa transmembrane glycoprotein involved in various cellular processes and is notably overexpressed in various solid tumours cancer cells^114^ and cancer stem cells.^115^ As such, hyaluronic acid has been used to post-synthetically modify the external surface of nanoMOFs and to provide targeting capabilities.^116–119^ Using a one-pot biomimetic mineralization process, Ding *et al.* used ZIF-8 to co-encapsulate chlorin e6 (a phososensitizer) and the enzyme cytochrome *c* before coating the external surface with hyaluronic acid.^119^ Importantly, both chlorin e6 and cytochrome *c* are larger than the porosity of ZIF-8, so the material grows around them. Using fluorescence microscopy and flow cytometry, the hyaluronic acid coating enabled active tumour targeting through the CD44 receptor binding in HeLa cells, and demonstrated *in vivo* tumour accumulation using fluorescence imaging in tumour-bearing mice. Another study based on ZIF-8 developed a one-pot synthesis to encapsulate doxorubicin; the system was then coated with polydopamine (to give the MOF an active surface), chelated with Fe^3+^ (for coordination) and conjugated with hyaluronic acid (for targeting): DOX@ZIF-HA.^118^ This multifunctional system had an enhanced intracellular uptake of DOX@ZIF-HA in PC-3 prostate cancer cells compared to mouse fibroblast L929 cells. However, the inclusion of DOX@ZIF nanoMOF control without HA modification *in vitro* would allow for further comparisons between this surface modification on cell uptake. Cai *et al.* demonstrated similar uptake properties using the Fe-based MIL-100 coated with hyaluronic acid for anticancer, photothermal therapy. Again, they showed enhanced cellular uptake in MCF-7 cells as well as intratumoral accumulation *in vivo* compared to MOFs without hyaluronic acid conjugation.^120^ However, an important caveat with any surface modification is that the addition of functionalisation changes the size, zeta potential and aggregation potential of particles which also can impact tumour cell uptake.

Whilst post-synthesis modifications with hyaluronic acid is a promising approach to targeted delivery, intracellular trafficking of hyaluronic acid nanoMOFs is still yet unknown. Indeed, the physicochemical and biological understanding of the trafficking mechanism is required to fully translate the material properties to biological applications. This would include, for example, *in vitro* studies characterising the binding dynamics of cell surface receptor expression (*e.g.* CD44) across the various cell models used in MOF studies. Additionally, systematically determining the physicochemical properties of the MOF particles and their coatings (*i.e.* charge, size, external surface density, hydrophobicity/hydrophilicity) with the inclusion of non-functionalized controls will enable sound comparisons of functionalization methods on endocytosis and trafficking. For example, the work by Qhattal and Liu investigated CD44-mediated uptake of hyaluronan-grafted liposomes in cancer cells by determining the effect of hyaluronan molecular weight, grafting density and CD44 receptor density on particle endocytosis.^121^ In this case, particle-CD44 binding and subsequent trafficking were impacted by the grafting density and hyaluronan chain length – showing that their hyaluronan-coated particles trafficked to low pH organelles. Similar properties will likely impact nanoMOF trafficking and, therefore, a more detailed biological evaluation would be advantageous.

### 4.4 nanoMOF camouflage: biological derived coatings

In recent years, attention has turned to novel biologically derived coating materials for nanoMOF external surface modification, such as exosomes and phospholipid cell membranes.^122,123^ Exosomes are 40 to 100 nm membrane-bound vesicles secreted by various, different cell types. They are reported to contain different components depending on their origin cell – including proteins, and nucleic acids^124^ – and play an important role in cellular signalling, both in healthy and diseased settings.^125,126^ Exosomes offer an advantage over synthetic coatings as, being naturally derived, they are likely to be non-immunogenic and readily recognized and trafficked into the cell, thus providing a natural camouflage. Because of these properties, they have shown promise in drug delivery as both carriers of drugs in their own right and as coatings of nanocarriers including nanoMOFs.^122,127,128^ The first example of this was a study using the fusion method to coat the surface of Fe-based MIL-88A with exosomes derived from HeLa cells.^122^ Coated particles loaded with the dye calcein were observed in HeLa cells following two days of incubation, with no reported premature cargo release observed. Although no internalization mechanism was reported, current studies suggest exosome uptake is largely *via* receptor binding, subsequent fusion and endocytosis. However, uptake is likely cell type- and context-dependent based on cell surface interactions.^129,130^

**Fig. 6:**
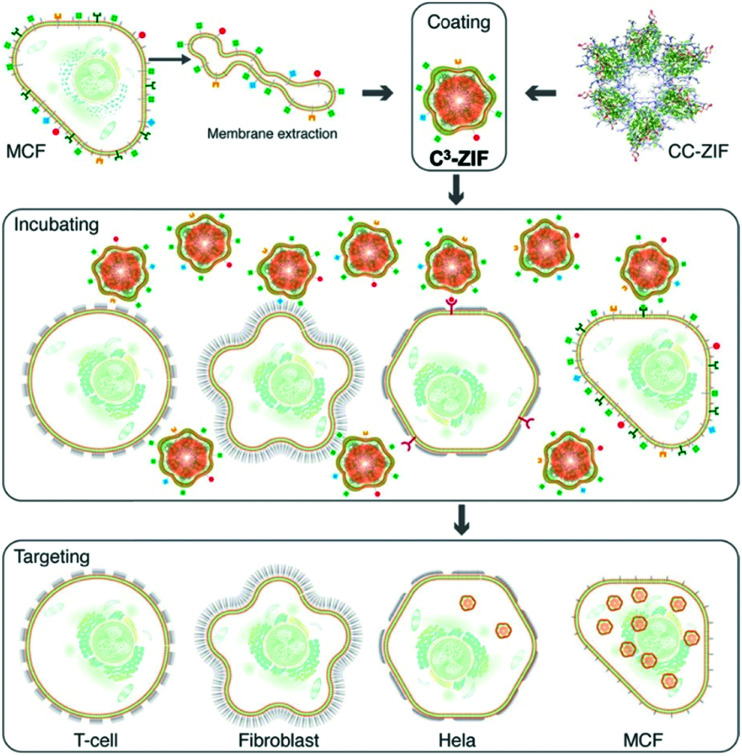
Schematic illustration of the preparation and cell-type selectivity of C^3^-ZIF. Human breast adenocarcinoma cells (MCF-7) were used as a model cancer cell line to coat CC-ZIF. The membrane-coated MOFs were then incubated with MCF-7, HeLa, fibroblast, and TC cell lines to study selective uptake. Reproduced from Alyami *et al.*^123^ with permission from American Chemical Society, copyright 2020.

In the case of cell phospholipids, Khashab and co-workers developed a biomimetic coating for ZIF-8 using cell membranes extracted from MCF-7 breast and HeLa cervical cancer cells. Using these cancer cell membranes (C), they managed to protect ZIF-8 and deliver clustered, regularly interspaced, short palindromic repeat (CRISPR) associated proteins 9 (Cas9), CRISPR/Cas-9 (CC), across MCF-7, HeLa, human dermal fibroblast, and T-lymphocyte cells (termed C^3^-ZIF MOFs, Fig. 6).^123^ In their case, the cell uptake of ZIF-8 was enhanced after specific coating: using ICP-MS, they observed a higher uptake of MCF-7-coated ZIF-8 in MCF-7 cells, and a higher uptake of HeLa-coated ZIF-8 in HeLa cells. Minimal internalization was observed in T-cell and HDF cells, notably reduced from the non-coated controls, indicating that coating reduced non-specific internalization. Interestingly, some lower levels of internalization were observed in HeLa cells with MCF-7-coated particles and *vice versa*, perhaps indicating some cancer-cell specific antigens present on both responsible for internalization – this is something that could be exploited in the future.

To develop the full potential of exosomes and membrane coatings in MOFs, it is critical to understand the routes of entry, characterizing the exact mechanisms of exosome- and cell membrane-coated MOF uptake. Indeed, although exosomes derived from cancer cells provide targeted treatment through fusing preferentially with their parent cancer cells,^131^ an important caveat is that cancer-derived exosomes are known to have tumour promoting properties, impacting cell growth, metastasis and motility, as well as reportedly affecting the properties of endothelial cells or stromal compartments.^132^ As such, using cancer-derived exosomes and cell membranes coatings for nanoMOF delivery could be a double-edged sword; using modified exosomes and cell membranes or those derived from non-cancerous cells may offer a safer strategy for improving nanoMOF stability, stealth properties and cellular uptake.

### 4.5 Protein corona

Detailed studies on the formation and impact of the protein corona on MOF uptake are limited, although speculation of the protein corona is a subject of discussion throughout the MOF literature. Indeed, the process of opsonization, whereby serum proteins bind to nanoparticle external surfaces and thus enable recognition and engulfment by macrophage cells is well-documented.^133^ Opsonization can also change the surface charge and particle size of nanoMOFs, thus impacting endocytic routes. Recent work carried out by Hidalgo and co-workers proposed a simple association strategy for the encapsulation of double-stranded siRNA using Fe-based MIL-100 and MIL-101-NH_2_.^62^ The intracellular delivery of cargo was assessed using human colorectal carcinoma cell line, SW480, and a fluorescent-labelled siRNA within the MOF, and measured using confocal fluorescence microscopy. They reported nanoMOF internalization into the cell cytoplasm and perinuclear areas. Analyses in serum-stimulated physiological media (containing FBS) saw an increase in nanoMOF particle size and aggregation. They also reported similar behaviour in supplemented cell culture media DMEM, showing larger particle sizes from 170 ± 69 to 253 ± 42 nm in MIL-100 (pH 4) and 287 ± 11 to 308 ± 96 nm in MIL-101 (pH 4). In addition to particle size, the protein corona also changes the electrostatic potential of materials. Both particle size and charge can influence the intracellular delivery and membrane interactions, which was evident in the difference in internalization between the two MOFs in this study, as internalization of siRNA@MIL-100 was significantly higher than siRNA MIL-101. Recent work by Zimpel *et al.* investigated the interaction of fluorescently tagged blood proteins with fluorescent Zr-fum MOF particles covered with various polymers using fluorescence cross-correlation spectroscopy. The selected proteins were albumin (Alb), the most abundant protein in human blood plasma, and immunoglobulin G (IgG), the most abundant type of antibody. The analysis showed a significant binding between fluorescent-IgG and both uncoated Zr-fum MOF NPs and Zr-fum MOF coated with negatively and positively charged polymers, while no binding was shown for albumin regardless of coating. When using a polysarcosine based copolymer, the interaction with IgG was much lower, leading to the conclusion that the coating is a determining factor for the interaction of Zr-fum NPs with different proteins.^134^ Gan *et al.* analyzed the protein corona formation on the MOF external surface using the Zr-based UiO-66 (257 nm) and UiO-67 (255 nm) with spherical and octahedral morphologies, respectively, in contact with human serum albumin (HSA).^135^ Protein adsorption and cell uptake studies using HeLa cells showed that the octahedral UiO-67 needed to bind with more HSA molecules to achieve a colloidal stability state than the spherical UiO-66, and this could affect material aggregation. It is not clear, though, how the surface chemistry of UiO-66 and -67 might play a role here. In any case, they demonstrated that the internalization of UiO-66 and UiO-67 by HeLa cells depended on the thickness of the protein corona. However, this was not foetal bovine serum (FBS) concentration-dependent as there was no increase in uptake when FBS concentration was elevated from 10% to 20%. This could perhaps be indicative of MOF saturation, or other biological factors at play. Further studies with ranges of FBS concentrations and timepoints would offer additional insight into this phenomenon.^135^ It is clear that more detailed studies about the formation of the protein corona on nanoMOFs are needed for accurate modelling of particle uptake, *in vitro* and *in vivo* behaviours, and subsequent downstream activity.

### 4.6 nanoMOF targeting using macromolecules

To date, there have been only a few studies incorporating a targeting moiety in the form of macromolecules on the MOF external surface to allow for receptor-specific targeted delivery. Chen *et al.* modified the external surface of an amino-triphenyl dicarboxylate-bridged a Zr-based nanoMOF with nucleic acids complementary to an aptamer against Vascular endothelial growth factor (VEGF) before loading it with doxorubicin and then capping with the VEGF aptamer.^139^ This created a VEGF-responsive nanoMOF with selective uptake – compared to MCF-10A control cells – into MDA-MB-231 breast cancer cells *via* aptamer AS1411 mediated nucleolin binding. Cherkassov *et al.* used the Fe-based nanoMOF MIL-100, also loaded with doxorubicin and coated with carboxymethyl dextran, and conjugated with the antibody trastuzumab through a hydrophilic carbohydrate interface.^136,137^ They demonstrated selective targeting of HER2 positive breast cancer cells *via* receptor-mediated uptake.^137^ Further scope for antibody-targeted MOFs was demonstrated in a study with Zn-based nanoMOFs that underwent post-synthetic modifications to incorporate an antibody against the tumour-associated glycoprotein epithelial cell adhesion molecule (EpCAM) to allow for selective tumour capture. Such methods could be adapted for targeted drug delivery through receptor-mediated mechanisms.^138^

### 4.7 Organelle targeting

In addition to tissue targeting, very recently, a number of studies have begun investigating the possibility of targeting MOF NPs to specific sub-cellular locations and organelles. Organelles are the fundamental functional units of the cell and as such, they are a critical target in several diseases. We have evaluated here the targeting of mitochondria, lysosomes and the nucleus of the cells.

#### 4.7.1 Mitochondria targeting

Mitochondria are the energy powerhouses of the cell. They are implicated in various biological processes, including metabolism, redox status maintenance, and regulation of cell survival and death.^139^ They play a key role in several disease states and thus constitute promising targets for novel treatments. Strategies to target mitochondria take advantage of their negative membrane potential, which is a lot higher than other organelles due to their role in ATP synthesis and proton-gradient maintenance.

A number of studies involving mitochondria-targeted MOFs have been reported since 2017, when Deng *et al.* described a mitochondria-targeted ZIF-90 for ATP imaging in live cells.^140–143^ They made use of the intrinsically high surface charge of ZIF-90 to target the negatively charged mitochondrial membrane. They encapsulated the fluorescent molecule Rhodamine-B (RhB) into ZIF-90, suppressing its emission. When ATP was present in the medium, it competitively coordinated with the Zn node of ZIF-90, disassembling it and releasing RhB for ATP sensing. The authors used this method to image mitochondria, the source of ATP production.^140^ Later, Dong and coworkers designed a targeted system based on ZIF-90 and heavy atom, iodine-attached BODIPY, to create 2I-BodipyPhNO_2_@ZIF-90.^142^ Again, they made use of the intrinsically high surface charge of ZIF-90 to target the negatively charged mitochondrial membrane. The system had high cell permeability, mitochondrial targeting ability, and strong 1O2-generating features. After incubation of MCF-7 and HepG2 cells with the system for 1 h under green light (540 nm, 20 mW cm^−2^), they detected significant apoptosis (15.8% and 14.7% of cells respectively) compared to almost no apoptosis in the control cells. This demonstrated an application of mitochondria-targeted MOFs in photodynamic therapy.

In addition to using the intrinsic positive surface charge of MOFs for mitochondria-targeting, several studies investigated the use of lipophilic cationic compounds such as triphenylphosphonium (TPP) as an effective mitochondria-targeting moiety. Haddad *et al.*, designed a mitochondria-targeting system by conjugating TPP to the zirconium-based MOF, UiO-66, to enhance the potency of dichloroacetate (DCA), an anti-cancer drug that acts on mitochondria. TPP was incorporated either during synthesis as a modulator or post-synthetically. Using super-resolution microscopy imaging, they showed the localization of the targeted system TPP@(DCA-UiO-66) in proximity to mitochondria, causing profound mitochondrial morphological changes, associated with cell death, as soon as 30 minutes after incubation. *In vitro* cell viability studies suggested that targeting DCA to mitochondria greatly reduced the amount required to less than 1% as compared to using the free drug, and less than 10% compared to the non-targeted system UiO-66-DCA. This study also expanded in the use of a whole-transcriptome analysis on MCF-7 cells, showing widespread changes in gene expression and especially in biological processes that have a profound effect on cell growth, metabolism, and survival. This work demonstrated how targeting MOFs to specific organelles can dramatically increase the efficacy and potency of existing drugs.^86^

Building on this, Arafa *et al.* covalently linked FA and TPP to the surface of the aminated Zr-based MOF, NH_2_-UiO-66, and successfully entrapped doxorubicin within its pores. A study of cell viability using the MTT assay demonstrated that the Dox-loaded dual-ligated NH_2_-UiO-66 displayed a higher anti-cancer efficiency towards hepatocellular carcinoma HepG2 cells. Flow cytometry assays showed that the mitochondria-targeting system triggered apoptosis and cell-cycle arrest more efficiently than the NPs coated with FA but not TPP. The work demonstrated the possibility of designing a MOF system to treat FA-overexpressing cancer types and cause mitochondria-mediated apoptosis.^144^ Gao *et al.* designed and synthesized a mitochondria-targeted biomimetic platinum nanozyme immobilized on a 2-D MOF, Sm-tetrakis(4-carboxyphenyl)porphyrin (Sm-TCPP), for photodynamic therapy. They assembled Sm^3+^ ions with TCPP, and then grew the catalase-mimicking platinum nanozymes *in situ*, forming Sm-TCPP-Pt. TPP was then attached to the system to give it mitochondrial targeting properties. The system was able to effectively convert the over-produced H_2_O_2_ in the tumor microenvironment of MCF-7 breast cancer cells into O_2_ to relieve tumor hypoxia. The generated reactive oxygen species near mitochondria significantly induced cell apoptosis.^145^ Wang *et al.* demonstrated the possibility of using a targeted MOF-based system as a catalyst to locally synthesize a drug in mitochondria. They constructed a MOF scaffold using the aminated Zr-based MOF NH_2_-UiO-66, into which they distributed ultra-fine copper nanoparticles. The composite was then modified with TPP to give it mitochondria-targeting properties (MOF-Cu-TPP). They tested the efficacy of their system by incubating MCF-7 cells with an inert prodrug of reservatrol, a drug with pro-apoptotic effects on mitochondria. The transformation of the prodrug to its active form occurred in the mitochondria and caused a considerable decrease in cell viability to 20%, whereas the survival rate of cells treated with pre-synthesized reservatrol alone was above 80% even at the highest tested concentration.^146^

#### 4.7.2 Lysosome targeting

Similar studies on lysosome targeting have been described. In particular, Zhao *et al.* designed and synthesized a lysosome-responsive NP, the acid-degradable ZIF-8, loaded with perforin and granzyme B, glycoproteins that lyse tumor cells and promote apoptosis respectively. They modified an CD63 aptamer with biotin on ZIF-8 to provide the system with lysosomal targeting abilities. Here, when the system reaches the acidic environment of lysosomes in T cells, ZIF-8 degrades and releases perforin and granzyme B, which remain stored in the lysosome until the T-cell receptor is activated by the major histocompatibility complex of tumor cells.^147^ All in all, this allows the creation of super-cytotoxic lymphocytes.

#### 4.7.3 Nucleus targeting

For the nucleus targeting, Liu and co-workers used a metal-oxide nanoenzyme derived from Mn-MOF for PDT.^148^ The particles showed good biocompatibility and high pore size and surface area, loading high doses of the photosensitizer chlorin e6 (Ce6). They modified the system using the nucleus-targeting AS1411 aptamer and PEG. The fabricated nanoenzyme possessed catalase-like activity, permitting the formation of O_2_ from H_2_O_2_, while consuming intracellular GSH. When irradiated with a 660 nm laser (200 mW cm^−2^, 5 min), the system produced abundant intracellular singlet oxygen radicals. In their case, they also observed reduced cell viability, demonstrating the cytotoxic activity of the system under normoxic conditions.^148^

## 5. Escaping the endosome trap

Endosomes are intracellular membrane-bound sorting organelles that are critical for a number of cellular processes, including important membrane trafficking processes. The term endosome escape is given when a material trafficked to the endosome can break free from the organelle and into the cytoplasm. Endosomal escape is an important strategy in the use of nanomaterials for drug delivery; it is especially relevant in terms of cargo transport, especially of nucleic acids, and is often known to be the rate-limiting step.^149–151^

Several studies have looked at ways to circumvent the endosomal trap. ZIF-8, extensively used in drug delivery, as shown in the above examples, has the benefit that the imidazole ligands work as a proton sponge, working effectively at the acid pH found and destroying the endosomal membrane. For example, Chen *et al.* reported the encapsulation and successful delivery of proteins using ZIF-8 nanoMOFs with a biocompatible polyvinylpyrrolidone (PVP) coating conferring extra hydrochemical stability.^152^ Using FITC-BSA encapsulated in the PVP-coated ZIF-8, they examined the intracellular delivery and endosomal escape in HeLa cells and showed that the nanoparticles were internalized within two hours. Using pharmacological inhibitors, they determined that the uptake was through lipid-raft mediated endocytosis. However, methyl-β-cyclodextrin – used here to inhibit lipid-raft mediated endocytosis – has been reported to be non-specific, with some studies showing impact on cell morphology and interfering with other endocytic pathways.^153^ All in all, it is possible that some of the NPs in this study were recycled out of the cell, rather than escaping the endosome, which would explain the low levels of localization with the lysosome. Here, further probing of the uptake and endosomal escape mechanism would provide some important insights into the productive trafficking of ZIF-8.

Another study using the ZIF-8 nanoMOF for the delivery of CRISPR/Cas-9 reported enhanced endosomal escape, leading to a 37% reduction in gene expression over 4 days.^154^ Fluorescence microscopy in CHO cells showed that, after one hour, the fluorescent Cas9 was localized inside endosomes but not after three and six hours later. They attributed this observation to the endosomal escape of ZIF-8 by protonation of the imidazole ring. However, in this case, endosomal localization was characterized using lysotracker green – a dye used to stain lysosomes but not endosomes. Further work would be useful to fully establish the mechanisms of escape for this system. Other work from Teplensky *et al.* showed the use of the Zr-based NU-1000 to deliver siRNA in HEK 293 cells. Whilst the NU-1000 was able to enter the cells, the downstream gene knockdown was variable and suboptimal when compared to the control using lipofectamine.^55^ To explore the hypothesis of endosomal trapping as the reason for the siRNA not being active, they included several co-factors such as a proton sponge, KALA peptide and ammonium chloride within the MOF system to aide endosomal escape. With the addition of the cofactors, there was a consistent level of gene knockdown, making clear that endosomal escape is a crucial part of the nanoMOF delivery story. Indeed, if the cargo does not reach its target destination, in this case in the cytosol, it will not show the desired effect. As well as establishing the physicochemical mechanisms to release nanoMOFs from the endosome in the future development of nanoMOFs as DDS, it will be important to find the pathways of uptake to avoid the endosomal trap.

## 6. Cell uptake and nanoMOF toxicity

The route of nanoMOF cellular uptake also has implications for MOF toxicity and immunogenicity. Despite a large number of publications on nanoMOFs for encapsulating and delivering therapeutics, there is a paucity of studies with in-depth cellular analysis of MOF eventual cellular fate or long term toxicity. To date a few investigations have examined the toxicity of MOFs in cellular, zebrafish embryo and *in vivo* models.^155–157^ One *in vitro* uptake study evaluating MOF metals and linkers in HeLa cells and murine macrophage cells showed Fe-based MOFs had a more favourable toxicity profile when evaluated with the MTT assay in these compared to Zr and Zn MOFs after 24 hour exposure.^156^ Wagner *et al.* evaluated MOFs ZIF-8 and MIL-160 in lung epithelial cells reporting lower toxicity in MIL-160 using real-time analysis, however of note in this study the particles were micron-sized and MOFs used in drug delivery applications are required to be on the nano scale for delivery applications^158^ Indeed, particle size not only has implications for the efficacy of delivery but also for cellular toxicity. Smaller sized particles (<50 nm) or too large have potential to cross the blood-brain barrier as demonstrated by Horcajada and colleagues who assessed the toxicity of MIL-88A, MIL-88B_4CH_3_ and MIL-100 in adult Wistar rats and found that the 40 nm MIL-88B_4CH_3_ was detectable in the brain tissue whereas the larger MIL nanoMOFs were not.^157^ Interestingly, they also reported differences in cellular uptake within the liver, showing that MOFs were present in Kupffer cells but not liver endothelial cells. Understanding cellular uptake mechanisms of nanoMOFs within the liver and spleen are also crucial for assessing MOF toxicity, and it would be interesting to explore if other nanoMOF materials with different surface modifications behave in a similar pattern. Also, and as mentioned previously particle aggregation is also an important consideration when evaluating the long term impact and fate of the MOF. Aggregation can affect the route of particle internalisation and interaction with the cellular membrane, as has been reported with non-functionalised MOFs^103^ it also has implications for immune cell uptake and phagocytosis of materials as larger particles are cleared more rapidly by the immune system.^159^ Understanding the trafficking and breakdown of nanoMOF within the cell is important for evaluating toxicity, as nanomaterials can form species such as reactive oxygen species which can lead to DNA damage upon degradation. An interesting study by Ploetz *et al.* showed that their lipid-modified Fe MIL-101 nanoMOF induced pyroptosis (programmed cell death *via* inflammation) in cells. This mechanism might likely be exploited by other nanoMOFs under similar conditions and further experimental studies with additional MOF materials would be interesting to explore. Ettinger *et al.* recently published a thorough review on the toxicity of MOF nanoparticles.^160^ Interestingly, they reported that, out of 95 papers, 70 used the MTT assay to evaluate toxicity. To build a full understanding of mechanisms of trafficking leading to cellular toxicity, other assays need to be used to build confidence in material behaviour and downstream effect within the cell. Time course or pulse-chase experiments would allow for *in vitro* modelling of stability, efficacy, and toxicity under different conditions.

The long-term fate of MOFs within cells is an important aspect of drug delivery that has received little attention to date. A large number of studies have indirectly addressed this issue by studying MOF degradation kinetics in different physiological buffers and at pH values that mimic the lumens of various intracellular compartments, namely late endosomes and lysosomes.^90,108,161^ Some studies have also exposed MOFs to biological proteases and assessed the resulting degradation.^162^ However, and to the best of our knowledge, only one study by Durymanov *et al.* has directly investigated the degradation dynamics and kinetics of MOFs inside a KUP5 cell.^87^ By observing individual MOF particles inside single cells using TEM, and by measuring the amount of iron released over time, they showed that the iron-based MIL-88B-NH_2_ and MIL-88A NPs accumulate in endolysosomes and start degrading within 15 minutes of incubation, reaching 10–15% decomposition after 24 h. Inhibiting phagosome acidification and protease activity did not prevent degradation, indicating a mode of degradation that is independent of pH and enzymatic activity. The mechanism of degradation and elimination of these iron-based MOFs remains elusive and yet to be determined. We highlight the need for more studies looking into the long-term fate of nanoMOFs inside cells.

## 7. Evaluating biological context: model and assay selection

Cell study is an essential part of the pharmacological characterization of nanoMOFs and, therefore, a thoughtful model selection is paramount to application and translational work. Indeed, detailed cellular analysis and endocytic profiling can increase our understanding of trafficking behaviours.^163,164^ As described previously, endocytosis is ubiquitous in mammalian cells but is regulated differently depending on the disease area, tissue and mutational landscape. Since cancer cells are derived from normal cells after the accumulation of mutations that endow them with selective advantages,^165^ endocytosis is one of the numerous physiological processes altered in them, driven by changes in protein expression and metabolic deregulation.

The mutational landscape of cancer cells can have a direct influence on cell uptake patterns and mechanisms of endocytosis, which can in turn influence nanoMOF delivery based on their particle size, morphology, and surface chemistry. One example of this is p53, a mutation hotspot that is inactivated in over half of human cancers.^166,167^ Wild type p53 is a tumor suppressor gene and it regulates cell cycle, DNA repair, metabolism and senescence.^167^ When mutated, p53 acts as a trans-dominant inhibitor of its wild type and contributes to malignant cellular activities such as invasion and metastasis.^167,168^ Mutated p53 stimulates invasive cell migration through promoting endocytic recycling of cell membrane receptors such as integrin and epithelial growth factor receptors.^168,169^ Turnover of these cell-surface receptors is associated with clathrin and caveolae-mediated uptake pathways.^170^ For example, the recycling of integrin α5β1 back to the membrane is controlled by GTPase Rab11 and Rab-coupling protein RCP – the process of which is inhibited by p63.^168,170^ However, mutated p53 overcomes this inhibitory effect, favors the endosome recycling over lysosome degradation, passes signals from pro-invasive kinase Akt, and enhances cancer invasiveness.^168^ Another example is the oncogenic RAS family, mutated across many oncogenic malignancies including non-small cell lung cancer (NSCLC), pancreatic adenocarcinoma (PDAC) and colorectal cancer (CRC), and is a subject of therapeutic targeting in itself.^171^ Studies of Ras-transformed PDAC cells showed upregulation of macropinocytosis to transport extracellular protein into the cell.^172^ Subsequent studies using albumin-bound NPs showed significant uptake of particles in KRAS mutant cells compared to wild type, indicating macropinocytosis mutation-driven mechanism of particle uptake.^173^ Cell uptake studies in HeLa cells and MCF-7 cells on two covalent organic frameworks (COFs) with comparable hydrodynamic size (120 nm) and zeta potentials (+13 mV) showed differences in cell uptake routes between the two cell types.^174^ It is clear that the rational selection of biological models is paramount as this will directly influence routes of nanomaterial uptake, and can be particularly informative when particle size and surface charge and chemistry are well controlled. To date, there have been numerous cell lines used to investigate the intracellular delivery of MOFs (Table 2). Most studies of MOFs for applications in cancer drug delivery utilize standard immortalized cancer cell models such as the cervical line HeLa and breast cancer cell lines MCF-7 and MDA-MB-231.^46,175,176^ Other immortalized cancer cell lines such as the glioblastoma cell lines U87MG and U251 have been used to investigate cellular trafficking nanoMOFs.^177,178^ For example, He *et al.* utilized amiloride, sucrose, and genistein as chemical inhibitors to investigate the uptake of UiO-66-H/N_3_, which they reported to be through clathrin- and caveolae-mediated pathways, and showed to co-localise with lysosomes. Whilst most studies have utilized immortalized cancer cell models in 2D systems, there are some interesting studies across other cell systems. For example, work with the fluorescently labelled Fe-based MIL-88B in Kupffer liver cells reported phagocytosis as the main route of internalization.^179^ The addition of primary patient models and more complex 3D or co-culture experiments will increase confidence in the tractability and translation of nanoMOFs for drug delivery purposes.

**Table 2:** Cell lines used in MOF studies

Ref.	Cell line	Description
83	HeLa	Cervical adenocarcinoma
180	MCF-7	Breast adenocarcinoma
45	MDA-MB-231	Breast adenocarcinoma
177	U87MG	Glioblastoma
181	4T1	*Mus musculus*; mimics stage IV human breast cancer
182	SCC7	Mouse squamous cell carcinoma
87	KUP5	Transformed murine Kupffer cell line
183	DC2.4	Transformed murine dendritic cell line
184	PC12	*Rattus norvegicus*; pheochromocytoma
176	3D4/21	*Sus scrofa*; alveolar macrophage cell lines
185	SKOV3-TR	Ovarian adenocarcinoma
186	RAW 264.7	Murine macrophage cell line
187	J774	Mouse reticulum cell sarcoma
178	U251	Glioblastoma
183	BMDCs	Bone marrow-derived dendritic cells from C57BL/6 mice
188	PAECs	Human pulmonary artery endothelial cells
	PASMCs	Human pulmonary artery smooth muscle cells
189	HUVECs	Human umbilical endothelial cells
190	MH-S	Murine alveolar macrophage

The selection of tools and the experimental design to evaluate nanoMOF uptake is one of the many challenges in tracking the endocytosis pathways. This remains a complex task, as most approaches have caveats with combinational strategies likeably to provide the most robust insights and avoid ambiguity. As mentioned in the previous section, small molecule inhibitors of endocytosis, commonly used to investigate nanoMOF trafficking, may be non-specific and have off-target side effects, meaning that conclusions from their use are limited.^191^ For example, clathrin inhibitor, Pitstop2, is reported to block many clathrin-dependent processes and is commonly used as a tool for understanding endocytic trafficking. However, studies in HeLa cells showed that it also inhibited cell growth by affecting spindle formation.^192^ It was also reported to block other pathways of endocytosis and has, therefore, been deemed as non-specific to clathrin.^193,194^ Hypertonic sucrose has also been widely used as a treatment to inhibit clathrin-mediated endocytosis. However, it has been reported to cause changes in the cell cytoskeleton.^195,196^ Likewise, inhibitors of macropinocytosis include rotterlin,^197^ amiloride derivatives,^198,199^ and rapamycin^199^ notably all have off-target effects and lack specificity towards macropinocytosis as a cellular trafficking process. Genistein is a tyrosine kinase inhibitor that is reported to inhibit caveolae-mediated endocytosis however, it also induces apoptosis and interferes in cellular processes by inducing autophagy in cancer cells.^200^ In addition, some inhibitors of endocytosis are also reported to cause cytotoxicity across some cell lines. For example, Braeckmans and co-workers showed that chlorpromazine (clathrin inhibitor) and methyl-beta-cyclodextrin (clathrin-independent inhibitor) significantly decreased cell viability across some cell lines at concentrations and timeframes often used in endocytic studies.^191^ In addition to inhibitors, the fluorescent markers used to track these endocytosis pathways or to determine the co-localisation of nanoMOF with specific organelles are also often problematic, often overlapping between their functions or providing ambiguous data. For example, fluorescent dextran is often used as a marker to study macropinocytosis in MOF studies. However, only at sizes of >70 kDa is this specific to macropinocytosis, and smaller formulations are likely to be taken up *via* clathrin and caveolin pathways.^201^ Another common method to study intracellular trafficking in real-time is by tagging the nanoMOF with a fluorescent marker. However some fluorescent tags can influence nanoparticle localisation, and if dyes become unconjugated from the nanoMOF this can cause misinterpretation of data. Here, the use of more in-depth biological techniques including gene-mediated knockdown (to study the impact of transient gene knockdown on nanoMOF trafficking pathways), or the use of clustered regularly interspaced short palindromic repeats (CRISPR) cell lines to study perturbations of specific endocytic pathways on nanoMOF uptake, might provide more robust insights into trafficking processes. Additionally, the use of advanced microscopy techniques such as structure illumination microscopy (SIM) and stimulated emission depletion (STED) microscopy for detailed resolution of intracellular distribution of nanoMOFs, together with high content confocal imaging platforms or flow cytometry or fluorescence-activated cell sorting (FACS) would provide statistical power as well as high resolution detail to enhance data interpretation.

Downstream cytotoxicity/viability assays can also prove to be challenging and limiting for evaluating nanoMOF efficacy. For example, the commonly used viability assays XTT (2,3-bis-(2-methoxy-4-nitro-5-sulfophenyl)-2*H*-tetrazolium-5-carboxanilide) and MTT (3-(4,5-dimethylthiazol-2-Yl)-2,5-diphenyltetrazolium Bromide)/MTS (3-(4,5-dimethylthiazol-2-yl)-5-(3-carboxymethoxyphenyl)-2-(4-sulfophenyl)-2*H*-tetrazolium) have issues with sensitivity and robustness.^202,203^ To build stronger foundations for the study of MOFs and nanomaterials in general, it is critical to evaluate the methods and tools one has at our disposal and work to establish more robust protocols and develop new techniques and approaches. Critical appraisal of well-established older methods, plus the inclusion of molecular biology techniques, stable CRISPR cell lines and various types of microscopy, flow cytometry and PCR could be used to provide a more detailed downstream understanding of nanoMOF behaviour.

## 8. Outlook

Undoubtedly, the field of nanoMOFs for drug delivery has advanced rapidly over the past decade, with research expanding from small molecule delivery to proteins and nucleic acids. Ever-evolving methods of MOF synthesis and design in terms of particle size, shape but also surface chemistry have led to exciting materials with sensing, theranostics and cell-specific targeting capabilities. However, with the rapid advancement of synthesis comes the need for biological scrutiny to enable the robust translation of these materials into clinical applications. While *in vitro* studies have evolved, there is still much room for progress – for example through the inclusion of appropriate controls to establish differences between on-target and off-target effects, thoughtful cell model selection, and examination of nanoMOFs in more complex cellular systems such as 3D systems, organoids and co-cultures. It is clear that understanding the cellular uptake and trafficking of nanoMOFs is a multifaceted process, with factors including particle physicochemical properties, surface modifications, biological models selected and assays used to evaluate behaviour – all having a role to play in building our understanding. Linking developments in MOF synthesis and their physicochemical properties with a biological understanding of the drug delivery process will offer exciting progress for the future.

## Abbreviations

α-CHC: α-Cyano-4-hydroxycinnamic acid
amiR: Artificial microRNA
API: Active pharmaceutical ingredient
ASO: Antisense oligonucleotides
Biot: Biotin
Cal: Calcein
CAU: Christian-albrechts-university
COF: Covalent organic framework
CRC: Colorectal cancer
DCA: Dichloroacetate
DDS: Drug delivery system
DMEM: Dulbecco's modified Eagle medium
DOX: Doxorubicin
FA: Folic acid
FACS: Fluorescence-activated cell sorting
FBS: Fetal bovine serum
Fol: Folate
FR: Folate receptor
5-FU: 5-Fluorouracil
HEK: Human embryonic kidney 293
HeLa: Henrietta Lacks (cervical cancer cell line)
ICP-MS: Inductively-coupled plasma mass spectrometry
IRMOF: Isoreticular MOF
MIL: Materials institute lavoisier
MOF: Metal–organic framework
mRNA: Messenger RNA
PCP: Porous coordination polymer
PCN: Porous coordination network
NP: Nanoparticle
NSCLC: Non-small cell lung cancer
NU: Northwestern university
PBL: Peripheral blood lymphocyte
PDAC: Pancreatic adenocarcinoma
PDT: Photodynamic therapy
PEG: Polyethylene glycol
RITC: Rhodamine B isothiocyanate
siRNA: Small interfering RNA
TLR: Toll-like receptor
UiO: Universitetet i Oslo
VER: Verapamil
ZIF: Zeolitic imidazolate framework
Zol: Zolendronate

## Conflicts of interest

D. F.-J. has a financial interest in the start-up company Vector Bioscience Cambridge, which is seeking to commercialize metal–organic frameworks.

## Supplementary Material
